# Supplementary material for: Outcomes of a bedside ultrasound-guided peripherally-inserted central catheter placement across critically-ill older patients
Source: PLoS One. 2026 Mar 20;21(3):e0336966. doi: 10.1371/journal.pone.0336966 (PMC13004389; doi:10.1371/journal.pone.0336966)
Supplement: Attachment — Submitted filename: Responses.doc [file pone.0336966.s003.doc]

PONE-D-25-11583

**An evidence-based protocol to control the bedside ultrasound-guided peripherally-inserted central catheter-related infection in elderly intensive-care unit patients in a small-volume center in Korea: A preliminary retrospective study**

PLOS ONE

Dear Dr. Lee,

Thank you for submitting your manuscript to PLOS ONE. After careful consideration, there are several formatting issues that need to be addressed before consideration for peer-review, with no guarantee of acceptance. I have outlined these below in order of the manuscript.

1. Abstract: Please provide a structured abstract, with headings such as Background, Methods, Results, and Conclusions, or as otherwise requested by the Journal. It is very difficult to understand the methods and background for this study from the abstract as it mainly focuses on results.

**[Responses]** We greatly appreciate your comments on our manuscript. As you have instructed, we revised the Abstract as follows:

“**Background** We performed a retrospective analysis of the data from a total of 241 patients (n=241).

**Methods** A total of 161 (66.8%) patients received the peripherally-inserted central catheter (PICC) for long-term intravenous access, 172 (71.4%) had no past history of receiving catheters and 142 (58.9%) received the PICC on the right side. Target veins include basilic vein (42.7% [103/241]), brachial vein (41.9% [101/241]) and cephalic vein (15.4% [37/241]).

**Results** There were a total of five cases (2.1%) of the PICC-related infection. Of these, one case (0.4%) was the PICC-related bloodstream infection; *Candida parapsilosis* was identified from both the PICC tip and blood samples. A total of 224 patients (92.9%) had optimal positions of the PICC tip. Male sex (OR 0.183; 95% CI 0.050-0.675, *p*=0.011), the length of a catheter (OR 0.794; 95% CI 0.657-0.960, *p*=0.017) and right side (OR 4.711; 95% CI 1.227-18.091, *p*=0.024) were significant risk factors of non-optimal positions of the catheter. Time-to-events are estimated at 56.02±1.37 days (95% CI 53.33-58.71). Moreover, survival rates are estimated to reach 0.909±0.044 (95% CI 0.826-0.999) at 29 days of the PICC use.

**Conclusions** Here, we describe our single-center, retrospective experience with bedside ultrasound (US)-guided PICCs in elderly ICU patients in a small-volume center.”

2. Introduction: The introduction is far too long (3.5 pages). This should be around 1 page, double spaced, with normal margins. Please make it more concise with a focus on relevant things without getting too detailed (i.e. the history of PICC, etc.). Other aspects can perhaps be placed in the Discussion if the authors feel is necessary.

**[Responses]** We greatly appreciate your comments on our manuscript.

We deleted the following statements:

“A central catheterization using a central venous catheter (CVC) or a peripherally-inserted central catheter (PICC) is referred to as an intravascular access to deliver injectate into a large central vein. Patients requiring medications that irritate smaller peripheral veins, parenteral nutrition and chemotherapies or the measurement of central venous pressure are indicated in a central catheterization.^[1]^”

“Since first introduced in 1975, it has gained popularity because of its advantages such as convenience of placement and cost-effectiveness.^[5,6]^ Indeed, it has become an integral part of treatment modalities in the realm of mainstream medicine.^[2]^ The use of PICC was approved by the United States Food and Drug Administration (FDA) for up to 3-12 months. After inserted, most of the PICCs are maintained at the site of placement for several months. But there is a variability in the actual period of use of PICC depending on its type, the method of insertion, a patient’s compliance and the technical expertise of a healthcare professional.^[7]^”

“The PICC has been conventionally performed under fluoroscopic guidance.^[8-10]^ But critically-ill patients may present with adverse effects during transport if they have respiratory failure or hemodynamic instability.^[11-13]^ In particular, intensive-care-unit (ICU) patients should run a risk while being transferred to other places because they are in need of specific management including diverse monitoring, continuous drug administration and ventilator support. According to a previous study, 37% of ICU patients presented with complications while being transferred to other places; the rate of such complications including worsening pulmonary function was approximately 2-fold higher as compared with normal controls.^[14,15]^ The safe use of PICC in unstable patients at the bedside would be mandatory. It can therefore be inferred that considerable efforts should be made to prevent the occurrence of such complications during transfer of critically-ill patients. From this context, attempts have been recently made to perform an ultrasound (US)-guided PICC at the bedside, thus termed as bedside US-guided PICC, for critically-ill patients.^[11,16-21]^”

PICC-related infections are classified as local exit-site infection, bacterial colonization and PICC-related BSI. Local exit-site infection is defined as the presence of erythema, induration and/or tenderness within 2 cm of the exit site, showing a positivity for the bacteria culture for swabs of exit-sites showed positive but being excluded from the possibility of contamination in accordance with the Infectious Diseases Society of America recommendations.^[22]^ Bacterial colonization is defined as the presence of ≥15 colony-forming units (CFUs) using the roll-plate technique or ≥1,000 CFUs by quantitative culture of pathogenic bacteria.^[39]^ PICC-related bloodstream infection (BSI) is defined as the presence of bacteremia arising from an intravenous catheter in the presence of clinical signs of infection, such as fever >38°C, chills, rigor and hypotension, and the absence of other sources of infection in accordance with the National Nosocomial Infections Surveillance System from the Centers for Disease Control and Prevention (CDC) (Atlanta, GA, USA) and from the Public Health Laboratory Service of the UK.^[22,40]^ It serves as the most common cause of nosocomial bacteremia.^[41]^ A diagnosis of PICC-related BSI can be made according to the Clinical Practice Guidelines for the Diagnosis and Treatment of Intravascular Catheter-related Infection.^[23]^ It can be established either based on the isolation of the same microorganism from ≥ one peripheral venous blood culture and from the catheter tip or from two blood culture samples each collected from a catheter hub and the peripheral vein.^[42]^ It is estimated that 16.4-28.8% of patients receiving the PICC are at a risk of developing PICC-related BSI; they are vulnerable to an increase in the mortality, morbidity and medical costs.^[22,43,44]^ Previous literatures have shown that there is a variability in the prevalence of PICC-related BSI, being estimated at 0.47-4.79 episodes per 1,000 catheter-days.^[45,46]^ More specifically, Maki DG, et al. estimated the prevalence of PICC-related BSI at 2.1 and 1.0 episodes per 1,000 catheter-days in hospitalized patients and outpatients, respectively.^[47]^”

3. Methods: Please place the Methods section directly after the Introduction and directly before the Results.

**[Responses]** We greatly appreciate your comments on our manuscript. We fixed all the formatting issues, as you have instructed.

4. Methods: Please add the STROBE checklist and mention this in the Methods: <https://www.strobe-statement.org/checklists/>

**[Responses]** We greatly appreciate your comments on our manuscript. We added the following statement to the Methods:

“The study was reported based on the STROBE checklist.”

5. Methods: It is unclear why mean and SD were used. I am concerned that the data distribution may be skewed. Please either provide data to suggest otherwise or consider median and IQR, along with non-parametric statistical tests

**[Responses]** We greatly appreciate your comments on our manuscript. In the current study, we used the mean and standard deviation rather than the median and interquartile range considering that there are no outliers in a study population.

6. Methods: As outlined in Table 5, please mention the exact regression technique in the methods (i.e. “logistic regression modelling was conducted...”) along with these covariates and how they were selected. Importantly, were they checked for colinearity? Please also provide the univariable, unadjusted estimates in a separate column for each variable (i.e. row).

**[Responses]** We greatly appreciate your comments on our manuscript. We already added the following statement to the Methods:

“Moreover, a logistic regression analysis was performed to identify risk factors of the non-optimal position of catheter tip.”

We also added the following statement:

“Before multiple regression analysis, multicollinearity was resolved by confirming variance inflation factors.”

7. Tables: There are far too many tables. Please consider combining or place some in a Supplementary File, alongside the STROBE checklist (as outlined in point 4 above)

**[Responses]** We greatly appreciate your comments on our manuscript. We merged former Tables 1, 2 and 4 as a single table as follows:

**Table 1. Baseline characteristics of the patients (n=241).**

| **Variables** | | | | | | | | | | | | | | | | | | | | **Values** |
| --- | --- | --- | --- | --- | --- | --- | --- | --- | --- | --- | --- | --- | --- | --- | --- | --- | --- | --- | --- | --- |
| **Age (years old)** | | | | | | | | | | | | | | | | | | | | 77.87±8.70 (66-99) |
|  | | | | | | 60-69 | | | | | | | | | | | | | | 53 (22.0%) |
|  |  |  |  |  |  | 70-79 | | | | | | | | | | | | | | 67 (27.8%) |
|  |  |  |  |  |  | 80-89 | | | | | | | | | | | | | | 103 (42.7%) |
|  |  |  |  |  |  | 90-99 | | | | | | | | | | | | | | 18 (7.5%) |
| **Sex** | | | | | | | | | | | | | | | | | | | | |
|  | | | | | | | | Men | | | | | | | | | | | | 129 (53.5%) |
|  |  |  |  |  |  |  |  | Women | | | | | | | | | | | | 112 (46.5%) |
| **SAPS2** | | | | | | | | | | | | | | | | | | | | 38.83±14.47 |
| **Status** | | | | | | | | | | | | | | | | | | | | |
|  | | | | | | | | Ventilator | | | | | | | | | | | | 95 (39.4%) |
|  |  |  |  |  |  |  |  | CRRT | | | | | | | | | | | | 40 (16.6%) |
| **Length of ICU stay (days)** | | | | | | | | | | | | | | | | | | | | 23.75±27.39 |
| **Length of hospital stay (days)** | | | | | | | | | | | | | | | | | | | | 39.89±41.88 |
| **Causes of ICU admission** | | | | | | | | | | | | | | | | | | | | |
|  | | | | | **CNS disorders** | | | | | | | | | | | | | | | |
|  |  |  |  |  |  | | | | | | | | Brain hemorrhage | | | | | | | 16 (6.6%) |
|  |  |  |  |  |  |  |  |  |  |  |  |  | Brain infarction | | | | | | | 9 (3.7%) |
|  |  |  |  |  |  |  |  |  |  |  |  |  | Infectious diseases | | | | | | | 3 (1.2%) |
|  | | | | | **CVS disorders** | | | | | | | | | | | | | | | |
|  |  |  |  |  |  | | | | | | | | HF | | | | | | | 27 (11.2%) |
|  |  |  |  |  |  |  |  |  |  |  |  |  | ACS | | | | | | | 10 (4.1%) |
|  |  |  |  |  |  |  |  |  |  |  |  |  | PTE | | | | | | | 2 (0.8%) |
|  |  |  |  |  |  |  |  |  |  |  |  |  | Infectious diseases | | | | | | | 2 (0.8%) |
|  |  |  |  |  |  |  |  |  |  |  |  |  | AD | | | | | | | 2 (0.8%) |
|  |  |  |  |  | **Pulmonary system disorders** | | | | | | | | | | | | | | | |
|  |  |  |  |  |  | | | | | | | | Pneumonia | | | | | | | 102 (42.3%) |
|  |  |  |  |  |  |  |  |  |  |  |  |  | COPD | | | | | | | 4 (1.7%) |
|  |  |  |  |  |  |  |  |  |  |  |  |  | Tuberculosis | | | | | | | 2 (0.8%) |
|  |  |  |  |  |  |  |  |  |  |  |  |  | Lung cancer | | | | | | | 6 (2.5%) |
|  |  |  |  |  | **Hepatobiliary system disorders** | | | | | | | | | | | | | | | |
|  |  |  |  |  |  | | | | | | | | | Liver cirrhosis | | | | | | 2 (0.8%) |
|  |  |  |  |  |  |  |  |  |  |  |  |  |  | rHCC | | | | | | 3 (1.2%) |
|  |  |  |  |  |  |  |  |  |  |  |  |  |  | Spleen injury | | | | | | 1 (0.4%) |
|  |  |  |  |  |  |  |  |  |  |  |  |  |  | Cholecystitis/Cholangitis | | | | | | 11 (4.6%) |
|  |  |  |  |  |  |  |  |  |  |  |  |  |  | Pancreatitis | | | | | | 1 (0.4%) |
|  |  |  |  |  | **Gastrointestinal system disorders** | | | | | | | | | | | | | | | |
|  |  |  |  |  |  | | | | | | | | Bleeding | | | | | | | 7 (2.9%) |
|  |  |  |  |  |  |  |  |  |  |  |  |  | Perforation | | | | | | | 11 (4.6%) |
|  |  |  |  |  |  |  |  |  |  |  |  |  | Obstruction | | | | | | | 3 (1.2%) |
|  |  |  |  |  |  |  |  |  |  |  |  |  | IBD | | | | | | | 2 (0.8%) |
|  |  |  |  |  | **Renal system disorders** | | | | | | | | | | | | | | | |
|  |  |  |  |  |  | | | | | | AKI | | | | | | | | | 15 (6.2%) |
| **Causes of ICU discharge** | | | | | | | | | | | | | | | | | | | | |
|  | | | | | | | Transfer to a ward | | | | | | | | | | | | | 138 (57.3%) |
|  |  |  |  |  |  |  | Transfer to other hospital | | | | | | | | | | | | | 35 (14.5%) |
|  |  |  |  |  |  |  | Death of a patient | | | | | | | | | | | | | 68 (28.2%) |
| **Causes of hospital discharge** | | | | | | | | | | | | | | | | | | | | |
|  | | | | | Return to home | | | | | | | | | | | | | | | 54 (22.4%) |
|  |  |  |  |  | Transfer to other hospital | | | | | | | | | | | | | | | 113 (46.9%) |
|  |  |  |  |  | Death of a patient | | | | | | | | | | | | | | | 74 (30.7%) |
| **Purposes of PICC** | | | | | | | | | | | | | | | | | | | | |
|  | | | | Long-term intravenous access | | | | | | | | | | | | | | | 161 (66.8%) | |
|  |  |  |  | Use of non-peripherally compatible infusate | | | | | | | | | | | | | | | 80 (33.2%) | |
| **The number of previous catheters** | | | | | | | | | | | | | | | | | | | | |
|  | | | 0 | | | | | | | | | | | | | | | | 172 (71.4%) | |
|  |  |  | ≥1 | | | | | | | | | | | | | | | | 69 (28.6%) | |
| **Side of PICC and the length of a catheter** | | | | | | | | | | | | | | | | | | | | |
|  | |  | | | | | | | Right side | | | | | | | | | | 142 (58.9%) | |
|  |  |  | | | | | | | | | | | | | | | Length (cm) | | 38.59±2.81 | |
|  |  |  | | | | | | | | Left side | | | | | | | | | 99 (41.1%) | |
|  |  |  | | | | | | | | | | | | | | | | Length (cm) | 43.58±3.30 | |
| **Target veins** | | | | | | | | | | | | | | | | | | | | |
|  | | | Brachial vein | | | | | | | | | | | | | | | | 101 (41.9%) | |
|  |  |  | Basilic vein | | | | | | | | | | | | | | | | 103 (42.7%) | |
|  |  |  | Cephalic vein | | | | | | | | | | | | | | | | 37 (15.4%) | |
| **Length of procedure time (min)** | | | | | | | | | | | | | | | | | | | 14.32±7.46 | |
| **Length of catheter indwelling time (days)** | | | | | | | | | | | | | | | | | | | 16.49±10.48 | |
| **Causes of PICC removal** | | | | | | | | | | | | | | | | | | | | |
|  | | Improvement of a patient’s condition | | | | | | | | | | | | | | | | | 93 (38.7%) | |
|  |  | Death of a patient | | | | | | | | | | | | | | | | | 51 (21.2%) | |
|  |  | Transfer to other hospital with PICC | | | | | | | | | | | | | | | | | 32 (13.3%) | |
|  |  | Accidental removal | | | | | | | | | | | | | | | | | 8 (3.3%) | |
|  |  | Replacement with new catheter | | | | | | | | | | | | | | | | | 31 (12.9%) | |
|  |  |  | | | | | | | | | | Suspicious catheter-related infection | | | | | | | 6 (2.5%) | |
|  |  |  |  |  |  |  |  |  |  |  |  | Non-function | | | | | | | 5 (2.1%) | |
|  |  |  |  |  |  |  |  |  |  |  |  | Swelling or hematoma | | | | | | | 11 (4.6%) | |
|  |  |  |  |  |  |  |  |  |  |  |  | Malposition | | | | | | | 4 (1.7%) | |
|  | **Locations of a catheter tip** | | | | | | | | | | | | | | | | | | | |
|  |  | | | | | | | **Optimal position** | | | | | | | | | | | | 224 (92.9%) |
|  |  |  |  |  |  |  |  | **Suboptimal position** | | | | | | | | | | | | |
|  |  |  |  |  |  |  |  |  | | | | | | | RA | | | | | 6 (2.5%) |
|  |  |  |  |  |  |  |  |  |  |  |  |  |  |  | Tributaries of the SVC | | | | | 5 (2.1%) |
|  |  |  |  |  |  |  |  | **Malposition** | | | | | | | | | | | | |
|  |  | | | | |  | | | | | | | | | | Ipsilateral IJV | | | | 2 (0.8%) |
|  |  |  |  |  |  |  |  |  |  |  |  |  |  |  |  | Ipsilateral SCV | | | | 1 (0.4%) |
|  |  |  |  |  |  |  |  |  |  |  |  |  |  |  |  | Arm vein | | | | 2 (0.8%) |

**Abbreviations:** SAPS2, Simplified Acute Physiology Score II; CRRT, continuous renal replacement therapy; ICU, intensive care unit; CNS, central nervous system; CVS, cardiovascular system; HF, heart failure; ACS, acute coronary syndrome; PTE, pulmonary thromboembolism; AD, aortic dissection; COPD, chronic obstructive pulmonary disease; rHCC, ruptured hepatocellular carcinoma; IBD, inflammatory bowel disease; AKI, acute kidney disease; PICC, peripherally-inserted central catheter; RA, right atrium; SVC, superior vena cava; IJV, internal jugular vein; SCV, subclavian artery.

Values are mean±standard deviation with range or the number of the patients with percentage, where appropriate.

8. Figures: Please add risk tables with events/total Ns to Figures 1 and 2. As per STROBE checklist, please also add a patient flow diagram that shows how patients were selected/how many excluded/how many finally included. Figure 3 should also ideally be proportion rather than number, please consider having the y-axis as %.

**[Responses]** We greatly appreciate your comments on our manuscript. We already provided Tables 4 and 5 to support two Kaplan-Meier graphs as follows:

**Table 4. Cumulative complication-free survival period.**

| **The total number of the patients** | **Incidences of the PICC-related infection** | **Censored value** | **TTEs (days)** |
| --- | --- | --- | --- |
| 241 | 5 | 236 (97.9%) | 56.02±1.37  (53.33-58.71) |

**Abbreviation:** PICC, peripherally-inserted central catheter; TTE, time-to-event.

TTEs are expressed as mean±standard error with 95% confidence intervals.

**Table 5. Cumulative hazards.**

| **Time points** | **n1** | **n2** | **Survival rates** |
| --- | --- | --- | --- |
| 13 | 144 | 1 | 0.993±0.006 (0.980-1.000) |
| 16 | 121 | 1 | 0.985±0.010 (0.964-1.000) |
| 28 | 51 | 1 | 0.966±0.021 (0.924-1.000) |
| 29 | 34 | 1 | 0.909±0.044 (0.826-0.999) |

**Note:** n1, the total number of the patients; n2, incidence of the peripherally-inserted central catheter-related infection.

Survival rates are expressed as mean±standard error with 95% confidence intervals.

We also added the following statement to the Results:

“From the study population, 36 patients aged 59 years or younger were excluded from the current study (Fig. 3).”

The disposition of the study patients was designated as Fig. 3.

Moreover, we revised or added the following two figures:

9. The study would also benefit from a close read and extensive typographical edits. For instance: 1) “A total of 241 patients (n=241)”. The (n=241) is unnecessary.

**[Responses]** We greatly appreciate your comments on our manuscript. We fixed all the typographical errors according to your instructions.

2) Rather than discussing granular age distributions, you can mention the mean or median (SD or IQR).

**[Responses]** We greatly appreciate your comments on our manuscript. In the current study, we used the mean and standard deviation rather than the median and interquartile range considering that there are no outliers in a study population.

3) Figures 1 and 2 have no numerical data and so it is not sufficient to just refer to them in the text. Please provide some numbers in the actual results.

**[Responses]** We greatly appreciate your comments on our manuscript. We already provided Tables 4 and 5 to support two Kaplan-Meier graphs as follows:

**Table 4. Cumulative complication-free survival period.**

| **The total number of the patients** | **Incidences of the PICC-related infection** | **Censored value** | **TTEs (days)** |
| --- | --- | --- | --- |
| 241 | 5 | 236 (97.9%) | 56.02±1.37  (53.33-58.71) |

**Abbreviation:** PICC, peripherally-inserted central catheter; TTE, time-to-event.

TTEs are expressed as mean±standard error with 95% confidence intervals.

**Table 5. Cumulative hazards.**

| **Time points** | **n1** | **n2** | **Survival rates** |
| --- | --- | --- | --- |
| 13 | 144 | 1 | 0.993±0.006 (0.980-1.000) |
| 16 | 121 | 1 | 0.985±0.010 (0.964-1.000) |
| 28 | 51 | 1 | 0.966±0.021 (0.924-1.000) |
| 29 | 34 | 1 | 0.909±0.044 (0.826-0.999) |

**Note:** n1, the total number of the patients; n2, incidence of the peripherally-inserted central catheter-related infection.

Survival rates are expressed as mean±standard error with 95% confidence intervals.

4) I am not sure why the results are all of a sudden summarized mid way through the Discussion. Ideally, the first paragraph of Discussion should provide a concise summary, then findings can be contextualized and discussed.

**[Responses]** We greatly appreciate your comments on our manuscript. We deleted the following statements:

“To summarize, our results are as follows:

(1) A total of 161 (66.8%) patients received the PICC for long-term intravenous access, 172 (71.4%) had no past history of receiving catheters and 142 (58.9%) received the PICC on the right side. Target veins include basilic vein (42.7% [103/241]), brachial vein (41.9% [101/241]) and cephalic vein (15.4% [37/241]).
(2) In our series, there were a total of five cases (2.1%) of the PICC-related infection. Of these, one case (0.4%) was the PICC-related BSI; *Candida parapsilosis* was identified from both the PICC tip and blood samples.
(3) A total of 224 patients (92.9%) had optimal positions of the PICC tip. A logistic regression analysis showed that male sex (OR 0.183; 95% CI 0.050-0.675, *p*=0.011), the length of a catheter (OR 0.794; 95% CI 0.657-0.960, *p*=0.017) and right side (OR 4.711; 95% CI 1.227-18.091, *p*=0.024) were significant risk factors of non-optimal positions of the catheter.

TTEs are estimated at 56.02±1.37 days (95% CI 53.33-58.71). Moreover, survival rates are estimated to reach 0.909±0.044 (95% CI 0.826-0.999) at 29 days of the PICC use.”

We added the following statement to the Discussion:

“Here, we describe our single-center, retrospective experience with bedside US-guided PICCs in elderly ICU patients in a small-volume center.”

5) Limitations should be in one cohesive paragraph and each point should be expanded upon (i.e. retrospective design, leading to the potential for misclassification and/or residual confounding). There are several other instances, including capitalization/grammatical changes needed.

**[Responses]** We greatly appreciate your comments on our manuscript. We revised the limitations as follows:

“Limitations of the current study are as follows: First, we conducted it under retrospective design in a small series of the patients who had been admitted to an ICU of a secondary referral center. Therefore, the possibility of selection bias could not be completely ruled out. Fourth, there was no control group in the current study. Second, we failed to analyze the learning curve of bedside ultrasound-guided PICCs in ICU patients in a small-volume center. It remains difficult to improve skills in performing bedside ultrasound-guided PICCs in ICU patients [52]. Nevertheless, there is a paucity of literatures showing the learning curve for acquisition of this capacity, although a recent study showed that the PICC is a well-established procedure with a small learning curve [77,78]. This is supported by Lassen K, et al. showing that the safety and efficacy of PICC are achieved without a learning curve [79]. Moreover, according to Kleidon TM, et al., it would be mandatory consider the variability in the learning curve in employing a new medical device into a healthcare service [80].”

10. The title is far too long. Consider something like: “Outcomes of a bedside ultrasound-guided peripherally inserted central catheter placement protocol across critically ill older adults: A retrospective study”

**[Responses]** We greatly appreciate your comments on our manuscript. We changed the Title into **Outcomes of a bedside ultrasound-guided peripherally inserted** **central catheter placement protocol across critically ill older adults: A retrospective study**, as you have instructed.

Please submit your revised manuscript as soon as possible or by May 12 2025

11:59PM. If you will need more time than this to complete your revisions, please reply

to this message or contact the journal office at [plosone@plos.org.](mailto:plosone@plos.org) When you’re ready to

submit your revision, log on to <https://www.editorialmanager.com/pone/>and select the

“Submissions Needing Revision” folder to locate your manuscript file.

Please include the following items when submitting your revised manuscript:

A rebuttal letter that responds to each point raised by the academic editor. You should upload this letter as a separate file labeled “Response to Reviewers”.

A marked-up copy of your manuscript that highlights changes made to the original version. You should upload this as a separate file labeled “Revised Manuscript with Track Changes”.

An unmarked version of your revised paper without tracked changes. You should upload this as a separate file labeled „Manuscript‟.

If you would like to make changes to your financial disclosure, please include your updated statement in your cover letter. Guidelines for resubmitting your figure files are available below the reviewer comments at the end of this letter.

If applicable, we recommend that you deposit your laboratory protocols in

protocols.io to enhance the reproducibility of your results. Protocols.io assigns your

protocol its own identifier (DOI) so that it can be cited independently in the future. For

instructions see: [https://journals.plos.org/plosone/s/submission-guidelines#loc-](https://journals.plos.org/plosone/s/submission-guidelines#loc-laboratory-protocols)

[laboratory-protocols](https://journals.plos.org/plosone/s/submission-guidelines#loc-laboratory-protocols). Additionally, PLOS ONE offers an option for publishing peer-

reviewed Lab Protocol articles, which describe protocols hosted on protocols.io. Read

more information on sharing protocols at

[https://plos.org/protocols?utm_medium=editorial-](https://plos.org/protocols?utm_medium=editorial-email&utm_source=authorletters&utm_campaign=protocols)

[email&utm_source=authorletters&utm_campaign=protocols.](https://plos.org/protocols?utm_medium=editorial-email&utm_source=authorletters&utm_campaign=protocols)

We look forward to receiving your revised manuscript.

Kind regards,

Kiyan Heybati

**Academic Editor**

PLOS ONE

**Journal requirements:**

When submitting your revision, we need you to address these additional requirements.

1. Please ensure that your manuscript meets PLOS ONE‟s style requirements, including those for file naming. The PLOS ONE style templates can be found at

[https://journals.plos.org/plosone/s/file?id=wjVg/PLOSOne_formatting_sample_main](https://journals.plos.org/plosone/s/file?id=wjVg/PLOSOne_formatting_sample_main_body.pdf) [_body.pdf](https://journals.plos.org/plosone/s/file?id=wjVg/PLOSOne_formatting_sample_main_body.pdf) and

[https://journals.plos.org/plosone/s/file?id=ba62/PLOSOne_formatting_sample_title_a](https://journals.plos.org/plosone/s/file?id=ba62/PLOSOne_formatting_sample_title_authors_affiliations.pdf) [uthors_affiliations.pdf](https://journals.plos.org/plosone/s/file?id=ba62/PLOSOne_formatting_sample_title_authors_affiliations.pdf)

2. Thank you for stating the following financial disclosure:

“This study was supported by a grant from the Jeju National University Hospital Research Fund (Grant No. 2019-35).”

Please state what role the funders took in the study. If the funders had no role, please state: “The funders had no role in study design, data collection and analysis, decision to publish, or preparation of the manuscript.”

If this statement is not correct you must amend it as needed.

Please include this amended Role of Funder statement in your cover letter; we will change the online submission form on your behalf.

**[Responses]** We greatly appreciate your comments on our manuscript. We added the following statement according to your instructions:

“But the funders had no role in study design, data collection and analysis, decision to publish, or preparation of the manuscript.”

3. For studies involving third-party data, we encourage authors to share any data specific to their analyses that they can legally distribute. PLOS recognizes, however, that authors may be using third-party data they do not have the rights to share.

**[Responses]** We greatly appreciate your comments on our manuscript. We added the following statement according to your instructions:

“**Data availability statement**

The datasets used and/or analysed during the current study available from the corresponding author on reasonable request.”

When third-party data cannot be publicly shared, authors must provide all information necessary for interested researchers to apply to gain access to the data. ([https://journals.plos.org/plosone/s/data-availability#loc-acceptable-data-access-restrictions](https://journals.plos.org/plosone/s/data-availability#loc-acceptable-data-access-restrictions))

For any third-party data that the authors cannot legally distribute, they should include the following information in their Data Availability Statement upon submission:

1) A description of the data set and the third-party source
2) If applicable, verification of permission to use the data set
3) Confirmation of whether the authors received any special privileges in accessing the data that other researchers would not have
4) All necessary contact information others would need to apply to gain access to the

data.

4. Please amend either the title on the online submission form (via Edit Submission) or the title in the manuscript so that they are identical.

[Note: HTML markup is below. Please do not edit.]

[NOTE: If reviewer comments were submitted as an attachment file, they will be attached to this email and accessible via the submission site. Please log into your account, locate the manuscript record, and check for the action link “View Attachments”. If this link does not appear, there are no attachment files.]

While revising your submission, please upload your figure files to the Preflight Analysis and Conversion Engine (PACE) digital diagnostic tool, [https://pacev2.apexcovantage.com/.](https://pacev2.apexcovantage.com/) PACE helps ensure that figures meet PLOS requirements. To use PACE, you must first register as a user. Registration is free. Then, login and navigate to the UPLOAD tab, where you will find detailed instructions on how to use the tool. If you encounter any issues or have any questions when using PACE, please email PLOS at [figures@plos.org.](mailto:figures@plos.org) Please note that Supporting Information files do not need this step.
