# Supplementary material for: Outcomes of a bedside ultrasound-guided peripherally-inserted central catheter placement across critically-ill older patients
Source: PLoS One. 2026 Mar 20;21(3):e0336966. doi: 10.1371/journal.pone.0336966 (PMC13004389; doi:10.1371/journal.pone.0336966)
Supplement: Attachment — Submitted filename: Responses 2.doc [file pone.0336966.s004.doc]

**Reviewers’ comments:**

**Reviewer’s Responses to Questions**

**Comments to the Author**

1. If the authors have adequately addressed your comments raised in a previous round of review and you feel that this manuscript is now acceptable for publication, you may indicate that here to bypass the “Comments to the Author” section, enter your conflict of interest statement in the “Confidential to Editor” section, and submit your “Accept” recommendation.

**Reviewer #1:** All comments have been addressed

**Reviewer #2:** (No Response)

**Reviewer #3:** All comments have been addressed

**Reviewer #4:** (No Response)

**Reviewer #5:** (No Response)

2. Is the manuscript technically sound, and do the data support the conclusions?

The manuscript must describe a technically sound piece of scientific research with data that supports the conclusions. Experiments must have been conducted rigorously, with appropriate controls, replication, and sample sizes. The conclusions must be drawn appropriately based on the data presented.

**Reviewer #1:** Yes

**Reviewer #2:** Partly

**Reviewer #3:** Partly

**Reviewer #4:** Yes

**Reviewer #5:** (No Response)

3. Has the statistical analysis been performed appropriately and rigorously?

**Reviewer #1:** Yes

**Reviewer #2:** I Don’t Know

**Reviewer #3:** Yes

**Reviewer #4:** Yes

**Reviewer #5:** (No Response)

4. Have the authors made all data underlying the findings in their manuscript fully available?

The PLOS Data policy requires authors to make all data underlying the findings described in their manuscript fully available without restriction, with rare exception (please refer to the Data Availability Statement in the manuscript PDF file). The data should be provided as part of the manuscript or its supporting information, or deposited to a public repository. For example, in addition to summary statistics, the data points behind means, medians and variance measures should be available. If there are restrictions on publicly sharing data—e.g. participant privacy or use of data from a third party—those must be specified.

**Reviewer #1:** Yes

**Reviewer #2:** Yes

**Reviewer #3:** Yes

**Reviewer #4:** Yes

**Reviewer #5:** (No Response)

5. Is the manuscript presented in an intelligible fashion and written in standard English?

PLOS ONE does not copyedit accepted manuscripts, so the language in submitted articles must be clear, correct, and unambiguous. Any typographical or grammatical errors should be corrected at revision, so please note any specific errors here.

**Reviewer #1:** Yes

**Reviewer #2:** Yes

**Reviewer #3:** Yes

**Reviewer #4:** Yes

**Reviewer #5:** (No Response)

6. Review Comments to the Author

Please use the space provided to explain your answers to the questions above. You may also include additional comments for the author, including concerns about dual publication, research ethics, or publication ethics. (Please upload your review as an attachment if it exceeds 20,000 characters)

**Reviewer #1:**

1-We therefore conducted this single-center, retrospective study to describe our protocol in a single small-volume center in an ICU setting in Korea. What do you mean protocol. What is the results of protocol. Protocol or outcome.

**[Responses]** We greatly appreciate your comments on our manuscript. We replaced the corresponding statement with the following one:

“We therefore conducted this single-center, retrospective study to describe outcomes of a bedside US-guided PICC across critically-ill older patients in a single small-volume center in an ICU setting in Korea.”

2- The patients underwent PICC placement under maximal barrier precautions using 2% chlorhexidine for skin preparation, surgical drapes, a surgical cap, sterile gown and gloves. 2% chlorhexidine alone or in combination with alcohol 70%.

**[Responses]** We greatly appreciate your comments on our manuscript. We revised the corresponding statement as follows:

“The patients underwent PICC placement under maximal barrier precautions, including the use of a surgical cap, sterile gown, sterile gloves, and large sterile drapes. For skin antisepsis, we used 2% chlorhexidine in 70% isopropyl alcohol.”

3-In our series, there were a total of five cases (2.1%) of the PICC-related infection. What is the definition of PICC infection in your study.

**[Responses]** We greatly appreciate your comments on our manuscript. We added the following phrase to the relevant part of the manuscript:

“, defined as a significant concern associated with the use of PICC. Such cases of infection may arise when bacteria or other germs enter the catheter and bloodstream, potentially leading to serious complications,”

**Reviewer #2:**

The inclusion and exclusion criteria were not well described. Equally, the study question was not well described.

**[Responses]** We greatly appreciate your comments on our manuscript. We already embedded inclusion/exclusion criteria in the Fig. 3, but added them to the manuscript text during revision as follows:

Inclusion criteria for the current study are as follows:

(1) Korean individuals
(2) ICU patients
(3) Elderly aged ≥60 years.

Exclusion criteria for the current study are as follows:

(1) The patients aged between 20 and 29 years old (n=5)
(2) The patients aged between 30 and 39 years old (n=7)
(3) The patients aged between 40 and 49 years old (n=14)
(4) The patients aged between 50 and 59 years old (n=31)
(5) The patients lost to follow-up (n=3)

The objectives of the study should be defined so it can guide the statistical analysis and the data comparison with the literature.

**[Responses]** We greatly appreciate your comments on our manuscript. We revised the study objectives as follows:

“We therefore conducted this single-center, retrospective study to describe outcomes of a bedside US-guided PICC across critically-ill older patients in a single small-volume center in an ICU setting in Korea.”

Guided by this information, researchers can better choose comparisons with the literature. In the manner of a more robust study.

**[Responses]** We greatly appreciate your comments on our manuscript. We added the following statements to the Discussion:

“In our series, there were nine cases (2.0%) of PICC-related infections. This is in agreement with a previous published report that the incidence of PICC-related exit-site infections is estimated at 1.9-60.9% [11]. PICC-related infections have a strong relationship with a length of hospital stay, ICU status, the number of device lumens, a previous PICC placement, an operator’s technical expertise, the time of catheter retention, the time of PICC indwelling, white blood cell counts, a history of diabetes mellitus and immunity [77-80]. Moreover, risk factors of PICC-related infections include patient-related factors (*e.g.*, chronic illnesses, immunosuppression and malnutrition) *as well as* catheter-related factors (*e.g.*, the time of PICC indwelling, catheter care delay and the site of PICC placement) [11].”

**Reviewer #3:**

1. Why sample stop at 2021? Is there any influence in determining the PICC procedure by ultrasound in the era of the Covid pandemic?

2. Is there any data above 2022 that can be added for data updates?

**[Responses]** We greatly appreciate your comments on our manuscript. We extended the length of study period between January of 2021 and December 2024. Then, we updated the data and revised the tables and figures according to your instructions as follows:

**Table 1. Baseline characteristics of the patients (n=452).**

| **Variables** | | | | | **Values** | |
| --- | --- | --- | --- | --- | --- | --- |
| **Age (years old)** | | | | | 77.89±8.67 (60-99) | |
|  | | 60-69 | | | 99 (21.9%) | |
|  |  | 70-79 | | | 126 (27.9%) | |
|  |  | 80-89 | | | 195 (43.1%) | |
|  |  | 90-99 | | | 32 (7.1%) | |
| **Sex** | | | | | | |
|  | | Men | | | 244 (54.0%) | |
|  |  | Women | | | 208 (46.0%) | |
| **SAPS2** | | | | | 39.27±14.45 | |
| **Status** | | | | | | |
|  | | Ventilator | | | 182 (40.3%) | |
|  |  | CRRT | | | 79 (17.5%) | |
| **Length of ICU stay (days)** | | | | | 24.38±27.79 | |
| **Length of hospital stay (days)** | | | | | 41.04±42.71 | |
| **Causes of ICU admission** | | | | | | |
|  | **CNS disorders** | | | | | |
|  |  | | Brain hemorrhage | | 30 (6.6%) | |
|  |  |  | Brain infarction | | 18 (4.0%) | |
|  |  |  | Infectious diseases | | 5 (1.1%) | |
|  | **CVS disorders** | | | | | |
|  |  | | HF | | | 52 (11.5%) |
|  |  |  | ACS | | | 20 (4.4%) |
|  |  |  | PTE | | | 4 (0.9%) |
|  |  |  | Infectious diseases | | | 4 (0.9%) |
|  |  |  | AD | | | 4 (0.9%) |
|  | **Pulmonary system disorders** | | | | | |
|  |  | | Pneumonia | | | 185 (40.9%) |
|  |  |  | COPD | | | 7 (1.5%) |
|  |  |  | Tuberculosis | | | 4 (0.9%) |
|  |  |  | Lung cancer | | | 11 (2.4%) |
|  | **Hepatobiliary system disorders** | | | | | |
|  |  | | Liver cirrhosis | | | 3 (0.7%) |
|  |  |  | rHCC | | | 6 (1.3%) |
|  |  |  | Spleen injury | | | 2 (0.4%) |
|  |  |  | Cholecystitis/Cholangitis | | | 22 (4.9%) |
|  |  |  | Pancreatitis | | | 2 (0.4%) |
|  | **Gastrointestinal system disorders** | | | | | |
|  |  | | Bleeding | | | 12 (2.7%) |
|  |  |  | Perforation | | | 22 (4.9%) |
|  |  |  | Obstruction | | | 6 (1.3%) |
|  |  |  | IBD | | | 4 (0.9%) |
|  | **Renal system disorders** | | | | | |
|  |  | | AKI | | | 29 (6.4%) |
| **Causes of ICU discharge** | | | | | | |
|  | | | Transfer to a ward | | | 258 (57.1%) |
|  |  |  | Transfer to other hospital | | | 66 (14.6%) |
|  |  |  | Death of a patient | | | 128 (28.3%) |
| **Causes of hospital discharge** | | | | | | |
|  | | | Return to home | | | 104 (23.0%) |
|  |  |  | Transfer to other hospital | | | 208 (46.0%) |
|  |  |  | Death of a patient | | | 140 (31.0%) |
| **Purposes of PICC** | | | | | | |
|  | | | Long-term intravenous access | | | 299 (66.2%) |
|  |  |  | Use of non-peripherally compatible infusate | | | 153 (33.8%) |
| **The number of previous catheters** | | | | | | |
|  | | | 0 | | | 318 (70.4%) |
|  |  |  | ≥1 | | | 134 (29.6%) |
| **Side of PICC and the length of a catheter** | | | | | | |
|  | | | Right side | | | 263 (58.2%) |
|  |  |  | Length (cm) | | | 38.53±2.82 |
|  |  |  | Left side | | | 189 (41.8%) |
|  |  |  | Length (cm) | | | 43.52±3.21 |
| **Target veins** | | | | | | |
|  | | | Brachial vein | | | 178 (39.4%) |
|  |  |  | Basilic vein | | | 203 (44.9%) |
|  |  |  | Cephalic vein | | | 71 (15.7%) |
| **Length of procedure time (min)** | | | | | | 14.20±7.33 |
| **Length of catheter indwelling time (days)** | | | | | | 16.56±10.58 |
| **Causes of PICC removal** | | | | | |  |
|  | | | Improvement of a patient’s condition | | | 172 (38.1%) |
|  |  |  | Death of a patient | | | 97 (21.5%) |
|  |  |  | Transfer to other hospital with PICC | | | 62 (13.7%) |
|  |  |  | Accidental removal | | | 16 (3.5%) |
|  |  |  | Replacement with new catheter | | | 56 (12.4%) |
|  |  |  |  | Suspicious catheter-related infection | | 12 (2.7%) |
|  |  |  |  | Non-function | | 9 (2.0%) |
|  |  |  |  | Swelling or hematoma | | 21 (4.6%) |
|  |  |  |  | Malposition | | 7 (1.5%) |
| **Locations of a catheter tip** | | | | | | |
|  | **Optimal position** | | | | | 421 (93.1%) |
|  | **Suboptimal position** | | | | |  |
|  |  | | RA | | | 10 (2.2%) |
|  |  |  | Tributaries of the SVC | | | 10 (2.2%) |
|  | **Malposition** | | | | |  |
|  |  | | Ipsilateral IJV | | | 3 (0.7%) |
|  |  |  | Ipsilateral SCV | | | 2 (0.4%) |
|  |  |  | Arm vein | | | 4 (0.9%) |

**Abbreviations:** SAPS2, Simplified Acute Physiology Score II; CRRT, continuous renal replacement therapy; ICU, intensive care unit; CNS, central nervous system; CVS, cardiovascular system; HF, heart failure; ACS, acute coronary syndrome; PTE, pulmonary thromboembolism; AD, aortic dissection; COPD, chronic obstructive pulmonary disease; rHCC, ruptured hepatocellular carcinoma; IBD, inflammatory bowel disease; AKI, acute kidney disease; PICC, peripherally-inserted central catheter; RA, right atrium; SVC, superior vena cava; IJV, internal jugular vein; SCV, subclavian artery.

Values are mean±standard deviation with range or the number of the patients with percentage, where appropriate.

**Table 2. Summary of cases of the peripherally-inserted central catheter-related infections.**

| **Variables** | | **Values** | | | |
| --- | --- | --- | --- | --- | --- |
| **N** | | 13 (2.88%) | | | |
| **Case #** | | Age  /Sex | Length of catheter indwelling time (days) | Pathogens isolated from the tip culture | Pathogens isolated from the blood culture |
|  | 1 | 91/M | 28 | *Staphylococcus capitis* | Negative |
|  | 2 | 66/M | 28 | *Candida carapsilosis* | *Candida parapsilosis* |
|  | 3 | 84/F | 14 | *Streptococcus mitis, S. oralis* and *Corynebacterium species* | Negative |
|  | 4 | 66/M | 28 | *Staphylococcus epidermidis* | Negative |
|  | 5 | 78/F | 16 | *Stenotrophomonas maltophilia* | Negative |
|  | 6 | 72/M | 21 | *Enterococcus faecali* | *Enterococcus faecali* |
|  | 7 | 88/F | 18 | *Klebsiella pneumoniae* | Negative |
|  | 8 | 65/M | 29 | *Pseudomonas aeruginosa* | *Pseudomonas aeruginosa* |
|  | 9 | 79/M | 25 | MRSA | MRSA |
|  | 10 | 87/F | 19 | ESBL-producing *Escherichia coli* | Negative |
|  | 11 | 93/M | 17 | *Candida albicans* | *Candida albicans* |
|  | 12 | 74/F | 20 | *Acinetobacter baumannii* | Negative |
|  | 13 | 69/M | 22 | *Serratia marcescens* | Negative |

**Note:** N, the total number of cases; F, female; M, male.

**Abbreviations:** MRSA, methicillin-resistant *Staphylococcus aureus*; ESBL, extended-spectrum β-lactamase.

Table 3. Risk factors of non-optimal positions of a catheter tip.

| **Variables** | **OR 95% CI** | ***p*-value** |
| --- | --- | --- |
| Age | 1.024 (0.976-1.074) | 0.330 |
| Male sex | 0.202 (0.078-0.521) | 0.001* |
| SAPS2 | 1.007 (0.978-1.037) | 0.628 |
| Long-term intravenous access | 0.665 (0.281-1.573) | 0.353 |
| Length of procedure time | 0.977 (0.917-1.04) | 0.462 |
| Length of a catheter | 0.786 (0.683-0.904) | 0.001* |
| Number of previous catheters | 1.994 (0.889-4.476) | 0.094 |
| Right side | 4.415 (1.649-11.824) | 0.003* |

**Note:** OR, odds ratio; CI, confidence interval.

**Abbreviation:** SAPS2, Simplified Acute Physiologic Score II.

*Statistical significance at *p*<0.05.

Table 4. Cumulative complication-free survival period.

| **The total number of the patients** | **Incidences of the PICC-related infection** | **Censored value** | **TTEs (days)** |
| --- | --- | --- | --- |
| 452 | 9 | 433 (98.0%) | 56.29±0.98  (54.37-58.21) |

**Abbreviation:** PICC, peripherally-inserted central catheter; TTE, time-to-event.

TTEs are expressed as mean±standard error with 95% confidence intervals.

Table 5. Cumulative hazards.

| **Time points** | **n1** | **n2** | **Survival rates** |
| --- | --- | --- | --- |
| 13 | 270 | 1 | 0.996±0.004 (0.989-1.000) |
| 14 | 259 | 1 | 0.992±0.005 (0.982-1.000) |
| 16 | 226 | 1 | 0.988±0.007 (0.975-1.000) |
| 20 | 172 | 1 | 0.982±0.009 (0.965-1.000) |
| 22 | 145 | 1 | 0.976±0.011 (0.954-0.998) |
| 27 | 108 | 1 | 0.967±0.014 (0.939-0.995) |
| 28 | 95 | 1 | 0.956±0.017 (0.923-0.991) |
| 29 | 65 | 1 | 0.942±0.022 (0.899-0.987) |
| 31 | 40 | 1 | 0.918±0.032 (0.858-0.983) |

**Note:** n1, the total number of the patients; n2, incidence of the peripherally-inserted central catheter-related infection.

Survival rates are expressed as mean±standard error with 95% confidence intervals.

**Fig. 1. Age distribution of the eligible patients.**

**Fig. 2. A peripherally-inserted central catheter (PICC).**

**Note:** V., vein.

**Fig. 3. Disposition of the study patients.**

**Fig. 4. Kaplan-Meier surivival.**

**Abbreviation:** PICC, peripherally-inserted central catheter.

In our series, time-to-events are estimated at 56.29±0.98 days (95% CI 54.37-58.21).

**Fig. 5. Kaplan-Meier cumulative hazards.**

Survival rates are estimated to reach 0.918±0.032 (95% CI 0.858-0.983) at 31 days of the peripherally-inserted central catheter use.

3. Elderly criteria has not been listed

**[Responses]** We greatly appreciate your comments on our manuscript. We already embedded inclusion/exclusion criteria in the Fig. 3, but added them to the manuscript text during revision as follows:

Inclusion criteria for the current study are as follows:

(4) Korean individuals
(5) ICU patients
(6) Elderly aged ≥60 years.

Exclusion criteria for the current study are as follows:

(6) The patients aged between 20 and 29 years old (n=5)
(7) The patients aged between 30 and 39 years old (n=7)
(8) The patients aged between 40 and 49 years old (n=14)
(9) The patients aged between 50 and 59 years old (n=31)
(10) The patients lost to follow-up (n=3)

4. In discussion please elaborate more about infection related to PICC

**[Responses]** We greatly appreciate your comments on our manuscript. We added the following statements to the Discussion:

“In our series, there were nine cases (2.0%) of PICC-related infections. This is in agreement with a previous published report that the incidence of PICC-related exit-site infections is estimated at 1.9-60.9% [11]. PICC-related infections have a strong relationship with a length of hospital stay, ICU status, the number of device lumens, a previous PICC placement, an operator’s technical expertise, the time of catheter retention, the time of PICC indwelling, white blood cell counts, a history of diabetes mellitus and immunity [77-80]. Moreover, risk factors of PICC-related infections include patient-related factors (*e.g.*, chronic illnesses, immunosuppression and malnutrition) *as well as* catheter-related factors (*e.g.*, the time of PICC indwelling, catheter care delay and the site of PICC placement) [11]”

5. What’s the novelty about USG guided PICC

**[Responses]** We greatly appreciate your comments on our manuscript. Please consider that we already provided the following statements in the Discussion:

“It is a general rule that the PICC is blindly inserted based on anatomical measurements of estimated distance [48]. The conventional use of anatomical landmarks provides the estimated length between the puncture site and the SVC-RA junction. Its advantages include relatively lower financial costs and acceptable performance. It can therefore be considered as the first-line of choice in patients with poor economic status. It is disadvantageous, however, in that patients are exposed to radiation and burdened with additional costs because of post-procedural chest X-rays [49]. Moreover, the success rate of the PICC based on the conventional use of anatomical landmarks is estimated at approximately 80%, which may not be regarded as a satisfactory outcome [2,50,51]. This explains why we routinely perform the PICC under US guidance in our institution. The utility of US guidance in bedside PICC has been advocated by several previous reports [52-56]. The success rate of conventional PICC varies, ranging from 65% to 75%, because there is a limited availability of the site of PICC (1.5 inches above to 1.5 inches below the antecubital fossa). But the success rate of US-guided PICC increases to 91-94% [1]. This is in agreement with our results showing that the rate of optimal position was 92.9% (224/241).”

6. The results section does not yet discuss risk factors of PICC related infection

**[Responses]** We greatly appreciate your comments on our manuscript. We added the following statements to the Discussion:

“In our series, there were nine cases (2.0%) of PICC-related infections. This is in agreement with a previous published report that the incidence of PICC-related exit-site infections is estimated at 1.9-60.9% [11]. PICC-related infections have a strong relationship with a length of hospital stay, ICU status, the number of device lumens, a previous PICC placement, an operator’s technical expertise, the time of catheter retention, the time of PICC indwelling, white blood cell counts, a history of diabetes mellitus and immunity [77-80]. Moreover, risk factors of PICC-related infections include patient-related factors (*e.g.*, chronic illnesses, immunosuppression and malnutrition) *as well as* catheter-related factors (*e.g.*, the time of PICC indwelling, catheter care delay and the site of PICC placement) [11]”

We also revised the Conclusions as follows:

“In conclusion, we describe outcomes of outcomes of a bedside US-guided PICC across critically-ill older patients in a single small-volume center in an ICU setting in Korea.”

**Reviewer #4:** This manuscript presents a study of 161 patients undergoing ultrasound-guided PICC placement, with findings suggesting that accurate tip positioning is critical for reducing PICC-related infections. After revision, the manuscript shows notable improvements in grammar and background framing, which enhance both the readability and the credibility of the conclusions.

However, several minor but important issues still require attention. Addressing these points would further improve the rigor and overall quality of the manuscript.

**[Responses]** We greatly appreciate your comments on our manuscript. First, we extended the length of study period between January of 2021 and December 2024. Then, we updated the data and revised the tables and figures according to your instructions as follows:

**Table 1. Baseline characteristics of the patients (n=452).**

| **Variables** | | | | | **Values** | |
| --- | --- | --- | --- | --- | --- | --- |
| **Age (years old)** | | | | | 77.89±8.67 (60-99) | |
|  | | 60-69 | | | 99 (21.9%) | |
|  |  | 70-79 | | | 126 (27.9%) | |
|  |  | 80-89 | | | 195 (43.1%) | |
|  |  | 90-99 | | | 32 (7.1%) | |
| **Sex** | | | | | | |
|  | | Men | | | 244 (54.0%) | |
|  |  | Women | | | 208 (46.0%) | |
| **SAPS2** | | | | | 39.27±14.45 | |
| **Status** | | | | | | |
|  | | Ventilator | | | 182 (40.3%) | |
|  |  | CRRT | | | 79 (17.5%) | |
| **Length of ICU stay (days)** | | | | | 24.38±27.79 | |
| **Length of hospital stay (days)** | | | | | 41.04±42.71 | |
| **Causes of ICU admission** | | | | | | |
|  | **CNS disorders** | | | | | |
|  |  | | Brain hemorrhage | | 30 (6.6%) | |
|  |  |  | Brain infarction | | 18 (4.0%) | |
|  |  |  | Infectious diseases | | 5 (1.1%) | |
|  | **CVS disorders** | | | | | |
|  |  | | HF | | | 52 (11.5%) |
|  |  |  | ACS | | | 20 (4.4%) |
|  |  |  | PTE | | | 4 (0.9%) |
|  |  |  | Infectious diseases | | | 4 (0.9%) |
|  |  |  | AD | | | 4 (0.9%) |
|  | **Pulmonary system disorders** | | | | | |
|  |  | | Pneumonia | | | 185 (40.9%) |
|  |  |  | COPD | | | 7 (1.5%) |
|  |  |  | Tuberculosis | | | 4 (0.9%) |
|  |  |  | Lung cancer | | | 11 (2.4%) |
|  | **Hepatobiliary system disorders** | | | | | |
|  |  | | Liver cirrhosis | | | 3 (0.7%) |
|  |  |  | rHCC | | | 6 (1.3%) |
|  |  |  | Spleen injury | | | 2 (0.4%) |
|  |  |  | Cholecystitis/Cholangitis | | | 22 (4.9%) |
|  |  |  | Pancreatitis | | | 2 (0.4%) |
|  | **Gastrointestinal system disorders** | | | | | |
|  |  | | Bleeding | | | 12 (2.7%) |
|  |  |  | Perforation | | | 22 (4.9%) |
|  |  |  | Obstruction | | | 6 (1.3%) |
|  |  |  | IBD | | | 4 (0.9%) |
|  | **Renal system disorders** | | | | | |
|  |  | | AKI | | | 29 (6.4%) |
| **Causes of ICU discharge** | | | | | | |
|  | | | Transfer to a ward | | | 258 (57.1%) |
|  |  |  | Transfer to other hospital | | | 66 (14.6%) |
|  |  |  | Death of a patient | | | 128 (28.3%) |
| **Causes of hospital discharge** | | | | | | |
|  | | | Return to home | | | 104 (23.0%) |
|  |  |  | Transfer to other hospital | | | 208 (46.0%) |
|  |  |  | Death of a patient | | | 140 (31.0%) |
| **Purposes of PICC** | | | | | | |
|  | | | Long-term intravenous access | | | 299 (66.2%) |
|  |  |  | Use of non-peripherally compatible infusate | | | 153 (33.8%) |
| **The number of previous catheters** | | | | | | |
|  | | | 0 | | | 318 (70.4%) |
|  |  |  | ≥1 | | | 134 (29.6%) |
| **Side of PICC and the length of a catheter** | | | | | | |
|  | | | Right side | | | 263 (58.2%) |
|  |  |  | Length (cm) | | | 38.53±2.82 |
|  |  |  | Left side | | | 189 (41.8%) |
|  |  |  | Length (cm) | | | 43.52±3.21 |
| **Target veins** | | | | | | |
|  | | | Brachial vein | | | 178 (39.4%) |
|  |  |  | Basilic vein | | | 203 (44.9%) |
|  |  |  | Cephalic vein | | | 71 (15.7%) |
| **Length of procedure time (min)** | | | | | | 14.20±7.33 |
| **Length of catheter indwelling time (days)** | | | | | | 16.56±10.58 |
| **Causes of PICC removal** | | | | | |  |
|  | | | Improvement of a patient’s condition | | | 172 (38.1%) |
|  |  |  | Death of a patient | | | 97 (21.5%) |
|  |  |  | Transfer to other hospital with PICC | | | 62 (13.7%) |
|  |  |  | Accidental removal | | | 16 (3.5%) |
|  |  |  | Replacement with new catheter | | | 56 (12.4%) |
|  |  |  |  | Suspicious catheter-related infection | | 12 (2.7%) |
|  |  |  |  | Non-function | | 9 (2.0%) |
|  |  |  |  | Swelling or hematoma | | 21 (4.6%) |
|  |  |  |  | Malposition | | 7 (1.5%) |
| **Locations of a catheter tip** | | | | | | |
|  | **Optimal position** | | | | | 421 (93.1%) |
|  | **Suboptimal position** | | | | |  |
|  |  | | RA | | | 10 (2.2%) |
|  |  |  | Tributaries of the SVC | | | 10 (2.2%) |
|  | **Malposition** | | | | |  |
|  |  | | Ipsilateral IJV | | | 3 (0.7%) |
|  |  |  | Ipsilateral SCV | | | 2 (0.4%) |
|  |  |  | Arm vein | | | 4 (0.9%) |

**Abbreviations:** SAPS2, Simplified Acute Physiology Score II; CRRT, continuous renal replacement therapy; ICU, intensive care unit; CNS, central nervous system; CVS, cardiovascular system; HF, heart failure; ACS, acute coronary syndrome; PTE, pulmonary thromboembolism; AD, aortic dissection; COPD, chronic obstructive pulmonary disease; rHCC, ruptured hepatocellular carcinoma; IBD, inflammatory bowel disease; AKI, acute kidney disease; PICC, peripherally-inserted central catheter; RA, right atrium; SVC, superior vena cava; IJV, internal jugular vein; SCV, subclavian artery.

Values are mean±standard deviation with range or the number of the patients with percentage, where appropriate.

**Table 2. Summary of cases of the peripherally-inserted central catheter-related infections.**

| **Variables** | | **Values** | | | |
| --- | --- | --- | --- | --- | --- |
| **N** | | 13 (2.88%) | | | |
| **Case #** | | Age  /Sex | Length of catheter indwelling time (days) | Pathogens isolated from the tip culture | Pathogens isolated from the blood culture |
|  | 1 | 91/M | 28 | *Staphylococcus capitis* | Negative |
|  | 2 | 66/M | 28 | *Candida carapsilosis* | *Candida parapsilosis* |
|  | 3 | 84/F | 14 | *Streptococcus mitis, S. oralis* and *Corynebacterium species* | Negative |
|  | 4 | 66/M | 28 | *Staphylococcus epidermidis* | Negative |
|  | 5 | 78/F | 16 | *Stenotrophomonas maltophilia* | Negative |
|  | 6 | 72/M | 21 | *Enterococcus faecali* | *Enterococcus faecali* |
|  | 7 | 88/F | 18 | *Klebsiella pneumoniae* | Negative |
|  | 8 | 65/M | 29 | *Pseudomonas aeruginosa* | *Pseudomonas aeruginosa* |
|  | 9 | 79/M | 25 | MRSA | MRSA |
|  | 10 | 87/F | 19 | ESBL-producing *Escherichia coli* | Negative |
|  | 11 | 93/M | 17 | *Candida albicans* | *Candida albicans* |
|  | 12 | 74/F | 20 | *Acinetobacter baumannii* | Negative |
|  | 13 | 69/M | 22 | *Serratia marcescens* | Negative |

**Note:** N, the total number of cases; F, female; M, male.

**Abbreviations:** MRSA, methicillin-resistant *Staphylococcus aureus*; ESBL, extended-spectrum β-lactamase.

Table 3. Risk factors of non-optimal positions of a catheter tip.

| **Variables** | **OR 95% CI** | ***p*-value** |
| --- | --- | --- |
| Age | 1.024 (0.976-1.074) | 0.330 |
| Male sex | 0.202 (0.078-0.521) | 0.001* |
| SAPS2 | 1.007 (0.978-1.037) | 0.628 |
| Long-term intravenous access | 0.665 (0.281-1.573) | 0.353 |
| Length of procedure time | 0.977 (0.917-1.04) | 0.462 |
| Length of a catheter | 0.786 (0.683-0.904) | 0.001* |
| Number of previous catheters | 1.994 (0.889-4.476) | 0.094 |
| Right side | 4.415 (1.649-11.824) | 0.003* |

**Note:** OR, odds ratio; CI, confidence interval.

**Abbreviation:** SAPS2, Simplified Acute Physiologic Score II.

*Statistical significance at *p*<0.05.

Table 4. Cumulative complication-free survival period.

| **The total number of the patients** | **Incidences of the PICC-related infection** | **Censored value** | **TTEs (days)** |
| --- | --- | --- | --- |
| 452 | 9 | 433 (98.0%) | 56.29±0.98  (54.37-58.21) |

**Abbreviation:** PICC, peripherally-inserted central catheter; TTE, time-to-event.

TTEs are expressed as mean±standard error with 95% confidence intervals.

Table 5. Cumulative hazards.

| **Time points** | **n1** | **n2** | **Survival rates** |
| --- | --- | --- | --- |
| 13 | 270 | 1 | 0.996±0.004 (0.989-1.000) |
| 14 | 259 | 1 | 0.992±0.005 (0.982-1.000) |
| 16 | 226 | 1 | 0.988±0.007 (0.975-1.000) |
| 20 | 172 | 1 | 0.982±0.009 (0.965-1.000) |
| 22 | 145 | 1 | 0.976±0.011 (0.954-0.998) |
| 27 | 108 | 1 | 0.967±0.014 (0.939-0.995) |
| 28 | 95 | 1 | 0.956±0.017 (0.923-0.991) |
| 29 | 65 | 1 | 0.942±0.022 (0.899-0.987) |
| 31 | 40 | 1 | 0.918±0.032 (0.858-0.983) |

**Note:** n1, the total number of the patients; n2, incidence of the peripherally-inserted central catheter-related infection.

Survival rates are expressed as mean±standard error with 95% confidence intervals.

**Fig. 1. Age distribution of the eligible patients.**

**Fig. 2. A peripherally-inserted central catheter (PICC).**

**Note:** V., vein.

**Fig. 3. Disposition of the study patients.**

**Fig. 4. Kaplan-Meier surivival.**

**Abbreviation:** PICC, peripherally-inserted central catheter.

In our series, time-to-events are estimated at 56.29±0.98 days (95% CI 54.37-58.21).

**Fig. 5. Kaplan-Meier cumulative hazards.**

Survival rates are estimated to reach 0.918±0.032 (95% CI 0.858-0.983) at 31 days of the peripherally-inserted central catheter use.

1. Issue in the Abstract – Background Section

The background section of the abstract is currently inadequate. Instead of providing scientific context, it merely describes the study itself. A well-constructed background should briefly introduce the clinical significance of the topic and highlight the specific scientific problem the study aims to address. In this case, the authors should synthesize key points from the Introduction: namely, that PICC is a commonly used alternative to central venous catheterization, but its application carries risks—particularly catheter-related bloodstream infections—which warrant further investigation. This study seeks to explore how tip location, side of insertion, and catheter length may influence such complications. The abstract should be revised to reflect this rationale clearly and concisely.

**[Responses]** We greatly appreciate your comments on our manuscript. We revised the Abstract of the manuscript as follows:

“An ultrasound (US)-guided peripherally inserted central catheter (PICC) is a thin, flexible tube inserted into a vein in the upper arm and then guided into a large vein near the heart, using US for precise vein location. We have devised an evidence-based protocol to control the bedside PICC-related infection in elderly intensive-care-unit (ICU) patients receiving bedside US-guided PICC in a small-volume center. We therefore conducted this single-center, retrospective study to describe outcomes of a bedside US-guided PICC across critically-ill older patients in a single small-volume center in an ICU setting in Korea.”

2. Right-Side PICC Placement for Patients Without Prior PICC – Methodological Clarification

In the Methods section, the authors mention a preference for right-sided PICC placement in patients without prior PICC history. While this is a retrospective study and procedures have already been performed, it is important to clarify whether this decision was evidence-based. For example, literature supporting right-sided preference in internal jugular central venous catheterization—due to anatomical landmarks, vessel course, or operator handedness—may be relevant. If such methodological justification exists, it should be cited explicitly.

Furthermore, a potential bias may be introduced by choosing right-sided placement only for patients without prior PICC insertion, who may have had better baseline health conditions compared to those who required previous catheterization. The authors should discuss how such selection bias was addressed or mitigated in their analysis.

**[Responses]** We greatly appreciate your comments on our manuscript. We added the following statements to the Discussion as you have instructed:

“In our retrospective cohort, the right-side PICC insertion for the patients with no prior PICC insertion involves inserting the catheter into a vein in the arm, typically the basilic, brachial or cephalic vein under US guidance, with the catheter advanced towards the SVC near the heart [31]. The right IJV is generally favored for PICC insertion due to its larger size and straighter path to the SVC, making it easier to access and potentially reducing complications. However, the left-sided IJV catheterization may be preferred in specific circumstances, and an operator’s handedness may affect the technique [81-83]. This is supported by peer-reviewed evidence based on anatomical advantages and lower rates of complications. In more detail, the right IJV is typically larger and has a more direct route to the SVC, making it easier to cannulate and potentially reducing a risk of complications, such as vessel injury or catheter malposition [83]. The right-sided IJV catheterization is associated with a lower rate of complications, such as carotid artery puncture, hematoma and pneumothorax as compared with the left-sided one [84-87].”

“Finally, the possibility of a potential bias arising from the right-sided preference in the patients with no prior PICC insertion could not be completely ruled out. It is likely that the patients with a history of PICC insertion may have had more severe underlying conditions or require more frequent infusions as compared with those without it. This causes differences in baseline health conditions and thereby could affect outcomes of the current study, with no respect to the PICC insertion line placement, which eventually makes it difficult to rule out differences in effects between the right-sided and left-sided cases [92].”

In conclusion, addressing these concerns would enhance the scientific integrity of the study and provide more robust evidence for optimizing ultrasound-guided PICC placement strategies to reduce complication rates related to suboptimal catheter positioning.

**Reviewer #5:** Thank you for the opportunity to review this manuscript. Unfortunately, I believe that the quality of this manuscript does not meet the standards of PLOS One, but this should not discourage the authors from continuing their valuable clinical research and thoroughly reviewing their manuscript before attempting submission elsewhere. Special attention should be given to better organisation of the structured abstract, and also to defining the research goals and hypothesis. It is unclear, but it looks like at least at one point the focus of this manuscript was infection control, in which case the developed protocol for infection control regarding placement, evaluation and maintenance of PICC should be discussed. Including an English language expert to proof-read the manuscript is advised.

**[Responses]** We greatly appreciate your comments on our manuscript. Please consider that the current study was conducted at a single, small-volume center in Korea. This is why we described its limitations as follows:

“Limitations of the current study are as follows: First, we conducted it under retrospective design in a small series of the patients who had been admitted to an ICU of a secondary referral center. Therefore, the possibility of selection bias could not be completely ruled out. Fourth, there was no control group in the current study. Second, we failed to analyze the learning curve of bedside US-guided PICCs in ICU patients in a small-volume center. It remains difficult to improve skills in performing bedside US-guided PICCs in ICU patients [52]. Nevertheless, there is a paucity of literatures showing the learning curve for acquisition of this capacity, although a recent study showed that the PICC is a well-established procedure with a small learning curve [88,89]. This is supported by Lassen K, et al. showing that the safety and efficacy of PICC are achieved without a learning curve [90]. Moreover, according to Kleidon TM, et al., it would be mandatory consider the variability in the learning curve in employing a new medical device into a healthcare service [91]. Finally, the possibility of a potential bias arising from the right-sided preference in the patients with no prior PICC insertion could not be completely ruled out. It is likely that the patients with a history of PICC insertion may have had more severe underlying conditions or require more frequent infusions as compared with those without it. This causes differences in baseline health conditions and thereby could affect outcomes of the current study, with no respect to the PICC insertion, which eventually makes it difficult to rule out differences in effects between the right-sided and left-sided cases [92].”

Also, please consider that this is a preliminary effort and encourage us to conduct further studies. Finally, we fixed language errors with the help of a professional writer from an English-speaking country. Again, we are very grateful for your instructions.

7. PLOS authors have the option to publish the peer review history of their article (what does this mean?). If published, this will include your full peer review and any attached files.

If you choose “no”, your identity will remain anonymous but your review may still be made public.

**Do you want your identity to be public for this peer review?** For information about this choice, including consent withdrawal, please see our Privacy Policy.

**Reviewer #1:** No

**Reviewer #2:** Yes: Felipe Martins Liporaci

**Reviewer #3:** No

**Reviewer #4:** No

**Reviewer #5:** No
