# Supplementary material for: Outcomes of a bedside ultrasound-guided peripherally-inserted central catheter placement across critically-ill older patients
Source: PLoS One. 2026 Mar 20;21(3):e0336966. doi: 10.1371/journal.pone.0336966 (PMC13004389; doi:10.1371/journal.pone.0336966)
Supplement: Attachment — Submitted filename: Responses 3.doc [file pone.0336966.s005.doc]

In addition to the reviewer comments below, please edit the Methods section of the Abstract to include more methodological items such as study dates, design, inclusion criteria, brief statistical analysis plans, etc. and the current information in the Methods of the Abstract can be condensed and perhaps moved to Results of the Abstract.

**[Responses]** We greatly appreciate your comments on our manuscript. We revised the Abstract according to your instructions as follows:

“**Background** An ultrasound (US)-guided peripherally inserted central catheter (PICC) is a thin, flexible tube inserted into a vein in the upper arm and then guided into a large vein near the heart, using US for precise vein location. We conducted this single-center, retrospective study to describe outcomes of a bedside US-guided PICC across critically-ill older patients in a single small-volume center in an intensive care unit (ICU) setting in Korea.

**Methods** We included 452 Korean older ICU patients aged ≥60 years who received PICC at our hospital between January of 2021 and December of 2024. A logistic regression analysis with odds ratio (OR) was performed to identify risk factors of the non-optimal position of catheter tip. The overall PICC-related infection-free survival was expressed as mean±standard error, for which 95% confidence intervals (CIs) were provided and the statistical significance was analyzed using the log-rank test. The corresponding Kaplan-Meier survival and hazards were plotted as a curve.

**Results** There were a total of 13 cases (2.88%) of the PICC-related infection. Of these, there were five cases (1.1%) of PICC-related bloodstream infection. A total of 421 patients (93.1%) had optimal positions of the PICC tip. A logistic regression analysis showed that male sex (OR 0.202; 95% CI 0.078-0.521, *p*=0.001), the length of a catheter (OR 0.786; 95% CI 0.683-0.904, *p*=0.001) and right side (OR 4.415; 95% CI 1.649-11.824, *p*=0.003) were significant risk factors of non-optimal positions of the catheter. Time-to-events are estimated at 56.29±0.98 days (95% CI 54.37-58.21). Moreover, survival rates are estimated to reach 0.918±0.032 (95% CI 0.858-0.983) at 31 days of the PICC use.

**Conclusions** We describe outcomes of a bedside US-guided PICC across critically-ill older patients in a single small-volume center in an ICU setting in Korea.”

As a minor point, please change “elderly” to “older adults.”

**[Responses]** We greatly appreciate your comments on our manuscript. We replaced “elderly” with “older adults”.

**Review Comments to the Author**

Please use the space provided to explain your answers to the questions above. You may also include additional comments for the author, including concerns about dual publication, research ethics, or publication ethics. (Please upload your review as an attachment if it exceeds 20,000 characters)

**Reviewer #2:** An important topic that demands attention from everyone who performs invasive procedures. The authors reviewed and responded to all suggestions from the review team. Based on this, the work ready for publication.

**[Responses]** We greatly appreciate your comments on our manuscript. Please encourage us to conduct further studies in this series.

**Reviewer #6:** Ultrasound is almost standard of care based on CDC and all other guidelines and should be used whenever available. The tip confirmation was still with CXR. So this is very standard operation.

<https://www.cdc.gov/infection-control/hcp/intravascular-catheter-related-infections/summary-recommendations.html>

Use ultrasound guidance to place central venous catheters (if this technology is available) to reduce the number of cannulation attempts and mechanical complications. Ultrasound guidance should only be used by those fully trained in its technique.

**[Responses]** We greatly appreciate your comments on our manuscript. We added the following statement to the relevant part of the manuscript:

“We used US guidance for PICC placement can reduce the number of attempts and mechanical complications, but ensured that it should only be performed by those with full training [24].”

Then, we added the following reference:

“Summary of Recommendations: Guidelines for the Prevention of Intravascular Catheter-Related Infections (2011). <https://www.cdc.gov/infection-control/hcp/intravascular-catheter-related-infections/summary-recommendations.html> Accessed October 21, 2025.”
